# Direct Cardiac Actions of Sodium-Glucose Cotransporter 2 Inhibition Improve Mitochondrial Function and Attenuate Oxidative Stress in Pressure Overload-Induced Heart Failure

**DOI:** 10.3389/fcvm.2022.859253

**Published:** 2022-05-12

**Authors:** Xuan Li, Elizabeth R. Flynn, Jussara M. do Carmo, Zhen Wang, Alexandre A. da Silva, Alan J. Mouton, Ana C. M. Omoto, Michael E. Hall, John E. Hall

**Affiliations:** Department of Physiology and Biophysics, Mississippi Center for Obesity Research, Mississippi Center for Heart Research, University of Mississippi Medical Center, Jackson, MS, United States

**Keywords:** empagliflozin, sodium-glucose cotransporter 2, heart failure, mitochondrial biogenesis, reactive oxidative species, apoptosis, autophagy

## Abstract

Clinical trials showed that sodium-glucose cotransporter 2 (SGLT2) inhibitors, a class of drugs developed for treating diabetes mellitus, improve prognosis of patients with heart failure (HF). However, the mechanisms for cardioprotection by SGLT2 inhibitors are still unclear. Mitochondrial dysfunction and oxidative stress play important roles in progression of HF. This study tested the hypothesis that empagliflozin (EMPA), a highly selective SGLT2 inhibitor, improves mitochondrial function and reduces reactive oxygen species (ROS) while enhancing cardiac performance through direct effects on the heart in a non-diabetic mouse model of HF induced by transverse aortic constriction (TAC). EMPA or vehicle was administered orally for 4 weeks starting 2 weeks post-TAC. EMPA treatment did not alter blood glucose or body weight but significantly attenuated TAC-induced cardiac dysfunction and ventricular remodeling. Impaired mitochondrial oxidative phosphorylation (OXPHOS) in failing hearts was significantly improved by EMPA. EMPA treatment also enhanced mitochondrial biogenesis and restored normal mitochondria morphology. Although TAC increased mitochondrial ROS and decreased endogenous antioxidants, EMPA markedly inhibited cardiac ROS production and upregulated expression of endogenous antioxidants. In addition, EMPA enhanced autophagy and decreased cardiac apoptosis in TAC-induced HF. Importantly, mitochondrial respiration significantly increased in *ex vivo* cardiac fibers after direct treatment with EMPA. Our results indicate that EMPA has direct effects on the heart, independently of reductions in blood glucose, to enhance mitochondrial function by upregulating mitochondrial biogenesis, enhancing OXPHOS, reducing ROS production, attenuating apoptosis, and increasing autophagy to improve overall cardiac function in a non-diabetic model of pressure overload-induced HF.

## Introduction

Although there have been improvements in the treatment and overall prognosis for heart failure (HF), the 5-year mortality rate is still high (1). Recently, sodium-glucose cotransporter 2 (SGLT2) inhibitors have been demonstrated to attenuate cardiac dysfunction and HF mortality in animal models and clinical trials (2–4). However, the mechanisms for cardiac protection by SGLT2 inhibitors are still unclear (5). Previous studies from our laboratory and from others have shown that empagliflozin (EMPA), a highly selective SGLT2 inhibitor, improves cardiac glucose and fatty acid oxidation, increases cardiac efficiency and improves overall cardiac function after transverse aortic constriction (TAC)-induced HF (6–8). However, the cellular mechanisms by which EMPA increases cardiac oxidative phosphorylation (OXPHOS) in failing hearts and improves cardiac function remain unclear. Mitochondria, the cell “energy factory,” supplies most of the energy needed by the heart. Thus, proper mitochondrial function is essential for maintaining a balance between cardiac energy supply and energy demand.

In HF, calcium mishandling and excessive oxidative stress can impair electron transport chain in mitochondria, leading to reduced fatty acid oxidation, uncoupling between glycolysis and glucose oxidation, and increased permeability of mitochondrial membranes. Release of reactive oxygen species (ROS), cytochrome C (Cyt C) and mitochondrial DNA (mtDNA) can induce inflammation, trigger apoptosis and result in cell death (9, 10). All these effects may further exacerbate HF. Conversely, clearance of damaged mitochondria through autophagy/mitophagy and attenuation of oxidative stress by endogenous antioxidants play cardioprotective roles in maintaining a healthy mitochondrial population. Thus, in the current study we tested the hypothesis that EMPA treatment may increase mitochondrial biogenesis, upregulate mitochondrial respiration and reduce ROS while favoring autophagy to protect the myocardium against TAC-induced cardiac dysfunction.

## Materials and Methods

### Animals

Male C57Bl/6J mice (3 to 4 months of age) were obtained from the Jackson Laboratory (Bar Harbor, ME). All experimental procedures and protocols conformed to the “Guide for the Care and Use of Laboratory Animals” published by the US National Institutes of Health. The animal protocols were approved by the Institutional Animal Care and Use Committee of University of Mississippi Medical Center. Mice were housed for at least 1 week under standard housing conditions before experiments, with a 12 h day/night cycle, and food and drinking water *ad libitum*. Mice were sacrificed by an overdose of 5% vol/vol isoflurane anesthesia (inhalation) followed by thoracotomy to harvest the heart.

### Transverse Aortic Constriction (TAC) Procedure

Mice were anesthetized with 2–3% vol/vol isoflurane, intubated and connected to a mechanical ventilator, and subcutaneously (SC) injected with bupivacaine (2 mg/kg) before surgical incision. Then a midline incision was performed in the anterior portion of the neck. The sternum was cut from the suprasternal notch down to the second intercostal space. The thymus was separated, the aortic arch was exposed, and a 7.0 silk suture was wrapped around the arch between the innominate artery and left carotid artery over a 27-gauge needle. The needle was then removed and the wound was closed (11). For the sham group, the procedure was similar, but without aortic constriction. After two weeks of TAC or sham surgery, the mice were given 200 μL of 1X phosphate buffered saline (PBS) as vehicle or EMPA (10 mg/kg/day by oral gavage) for the duration of the study which lasted an additional 4 weeks.

### Body Weight and Fasting Blood Glucose Measurements

Body weight and fasting blood glucose were measured at baseline and the end of the protocol, respectively. The mice were fasted for 6 h (8:00 a.m. to 2:00 p.m.) and blood glucose levels were determined using a glucose meter (ReliOn, USA).

### Echocardiography

Animals from each group were anesthetized with 1–2% isoflurane (inhalation) and transthoracic echocardiography (Vevo 3100, Visualsonics, Toronto, Canada) was performed to measure cardiac function, including systolic and diastolic parameters. Data from at least three cardiac cycles were collected. Left ventricular (LV) trace was performed to obtain an averaged ejection fraction (EF) and fraction shortening (FS) for each group. Diastolic function was measured by PW Doppler imaging including isovolumetric relaxation time (IVRT), isovolumetric contraction time (IVCT), and ejection time (ET). Myocardial performance index (MPI) was calculated as: (IVCT + IVRT)/ET.

### Left Ventricular Pressure-Volume Loop Analysis

At the end of the protocol, left ventricular pressure-volume loop analysis was performed as previously described (12). Briefly, the mice were anesthetized with urethane (1 g/kg, i.p.). After implanting a (RenaPulse tubing, 120 RPT040, Braintree, MA) catheter in the internal jugular vein for hypertonic saline bolus injections, a catheter (Millar 1.4F, SPR 839, Millar Instruments) was inserted into LV through the apex. Hypertonic saline (15% NaCl, 5 μl) was injected to obtain parallel conductance. The following parameters were obtained: heart rate (HR), maximum dP/dt (+dP/dtmax), minimum dP/dt (–dP/dtmin), isovolumetric relaxation time constant (tau), end-diastolic pressure volume relationship (EDPVR), and LV end-diastolic pressure (LVEDP).

### Transmission Electron Microscopy

Heart tissues were rapidly immersed in tissue fixative buffer (10% formaldehyde, buffered, pH 7.4, Carson-Millonig formulation; RI31911; Ricca Chemicals, Arlington, TX, USA) at 4°C for at least 8 h. Fixed tissues were trimmed to 1 mm^3^, stained with OsO4 for 1 h, dehydrated in a graded ethanol series for 10 min per step (once in 35, 50, 70, and 95% and twice in 100% ethanol), washed twice with acetone for 15 min per wash, washed in a solution of 1:1 acetone:Epon for 1 h, and finally embedded in 100% Epon (Ted Pella, Redding, CA, USA) for 30 min. After incubating at 60°C overnight, the Epon block was semithin sectioned (10–12 1-μm-thick sections) by using Sorvall MT-6000-XL (RMC Boeckeler, Tucson, AZ, USA) and a glass knife. Semithin sections were stained with 1% toluidine and observed under a light microscope to locate areas of interest for ultrathin sections. After trimming, the Epon block was thin sectioned (70 nm thick) by using a Leica Reichert Ultracut microtome and diamond knife. Thin sections were then applied on copper grids, air dried, stained with 2% uranyl acetate for 3 min and calcinated lead citrate for 30 s, and rinsed in distilled water by briskly dipping up and down for 20 s. The stained grid was loaded in a Jem1400 transmission electron microscopy (Jeol, Tokyo, Japan) with an ANT camera system. At least 5 sections from each sample were examined with the transmission electron microscope. Each entire section was thoroughly viewed at low magnifications to find areas of interest and to observe size, shape and arrangement of mitochondria.

### Immunohistochemistry

Heart paraffin embedded tissue sections (5 μm) were prepared as described previously (11). After they were dewaxed and rehydrated, the sections were treated with 0.1 M sodium citrate buffer for antigen retrieval. Then they were incubated with 10% normal donkey serum for 20 min at room temperature. Sections were then incubated at 4°C overnight with cleaved caspase-3 (Cell Signaling, 14220) (1:500 dilution). Sections were rinsed in PBS and incubated with a horseradish peroxidase (HRP) secondary antibody (Cell Signaling, 8114) in block solution (1:500 dilution) and DAB (3,3'-diaminobenzidine) (Cell Signaling, 8059) at room temperature. Following PBS rinse, sections were counterstained sections with hematoxylin (Cell Signaling, 14166). Finally, these sections were observed under the light microscope.

### Mitochondrial/Cytosol Fractionation

The separation of mitochondria and cytosol in hearts was performed following the protocol detailed by the manufacturer (Abcam, ab65320).

### Immunofluorescence Staining

The cardiac sections for immunofluorescence staining were prepared as described for immunohistochemistry. Then, the sections were incubated at 4°C overnight following addition of Cyt C (1:500, Cell Signaling, 12963) and IraZolve-Mito (1:1000, Cayman, 25910). Subsequently, samples were washed three times and incubated with fluorescent secondary antibodies (goat anti-rabbit IgG, 1:500, Alexa Fluor® 555, A32732) at room temperature for 2 h in the dark wet chamber. Samples were washed with PBS, stained with DAPI for 5 min at room temperature, mounted, and then observed under fluorescence microscope.

### Masson and Terminal Deoxynucleotidyl Transferase dUTP Nick End Labeling (TUNEL) Staining

Masson trichrome stain (Sigma-Aldrich) and TUNEL staining (Cell Death Detection Kit, Roche, 11684809910) were performed following the protocol detailed by the manufacturer.

### Immunoblotting

Immunoblotting was performed as previously described (13–15). Briefly, protein concentration was measured using the Bradford dye-binding method (Dye Reagent Concentrate, Bio-Rad Protein Assay). The target proteins were separated by SDS-PAGE and transferred to Millipore nitrocellulose membranes (BioRad, Hercules, CA). The membranes were incubated with primary antibodies at 4°C overnight. Then, membranes were incubated with secondary antibodies at room temperature for 1 h and signal intensity was determined using the Odyssey Infrared Imaging System (LI-COR, Lincoln, NE).

Antibodies against phospho-AMP-activated protein kinase (p-AMPK) (Cell Signaling, 2535), AMPK (Cell Signaling, 5831), phospho-mammalian target of rapamycin (p-mTOR) (Cell Signaling, 2971), mTOR (Cell Signaling, 2983), phospho-Unc-51 like autophagy activating kinase (p-Ulk1) (Cell Signaling, 14202), Ulk1 (Cell Signaling, 8054), autophagy related 7 (Atg7) (Cell Signaling, 8558), Beclin1 (Novus, NB500-249), light chain 3 (LC3) A/B (Cell Signaling, 4108), Bcl-2 (Cell Signaling, 3498), Bax (Cell Signaling, 14796), cleaved caspase-3 (Cell Signaling, 9664), phospho-extracellular signal-regulated kinase (p-ERK) (Cell Signaling, 4370), ERK (Cell Signaling, 4695), phospho-Bad (Cell Signaling, 9291), Bad (Cell Signaling, 9292), nuclear respiratory factor 1 (NRF-1) (Novus, NBP2-75597), the nuclear factor erythroid 2–related factor 2 (NRF-2) (Novus, H00004780-M02), heme oxygenase-1 (HO-1) (Cell Signaling, 86806), peroxisome proliferator-activated receptor gamma coactivator 1-alpha (PGC1-α) (Novus, NBP1-04676), mitochondrial transcription factor A (TFAM) (Novus, NBP2-19437), cyclooxygenase1 (COX1) (Cell Signaling, 9896), glyceraldehyde 3-phosphate dehydrogenase (GAPDH) (Cell Signaling, 2118), were used as primary antibodies. IRDye® 800CW Donkey anti-Rabbit IgG (LI-COR, 925-32213) and IRDye® 680RD Donkey anti-Rabbit IgG (LI-COR, 925-68073) were used as secondary antibodies.

### Real-Time Quantitative Polymerase Chain Reaction (RT-qPCR)

Total RNA was isolated from heart tissues using Trizol reagent (Invitrogen,15596026) and real-time qPCR was performed as previously described (14). RT-qPCR was performed in a 10 μL reaction mixture prepared with SYBR GREEN PCR Master Mix (Applied Biosystems, Warrington, UK) containing an appropriately diluted cDNA solution and 0.2 mM of each primer at 95°C for 10 min, followed by 35 cycles at 95°C for 10 s and 60°C for 45 s. All reactions were conducted in triplicate, and data were analyzed using the delta Ct (ΔΔCt) method. These transcripts were normalized to β-actin. The details of primers are shown in Table 1.

**Table 1:** RT-qPCR primers.

**Gene**	**Species**	**Forward**	**Reverse**
*Atg7*	Mouse	5'-CCTGTGAGCTTGGATCAAAGGC-3'	5'-GAGCAAGGAGACCAGAACAGTG-3'
*Beclin1*	Mouse	5'-GGCCAATAAGATGGGTCTGA-3'	5'-GCTGCACACAGTCCAGAAAA-3'
*Catalase*	Mouse	5'-TTGGTGCCTTGGTCACTGTGTTAG-3'	5'-GACTGGAATGCTTCGTGGCTCTC-3'
*COX1*	Mouse	5'-ATCACTACCAGTGCTAGCCG-3'	5'-CCTCCAGCGGGATCAAAGAA-3'
*GAPDH*	Mouse	5'-GGTTGTCTCCTGCGACTTCA-3'	5'-TGGTCCAGGGTTTCTTACTCC-3'
*GCLM*	Mouse	5'-CTGGCTGTCCTGGAACTCACTTTG-3'	5'-GAGGCGGAGGCAGAGGTAGAG-3'
*HO-1*	Mouse	5'-CCTGCTGATTCTCCTCTCCTCCTC-3'	5'-AAGCCTTCTCTGGACACCTGACC-3'
*LC3*	Mouse	5'-GACGGCTTCCTGTACATGGTTT-3'	5'-TGGAGTCTTACACAGCCATTGC-3'
*NRF-1*	Mouse	5'-GCACCTTTGGAGAATGTGGT-3'	5'-GGGTCATTTTGTCCACAGAGA-3'
*NRF-2*	Mouse	5'-CTGTGCTGCCAGAGGTCCTTAATG-3'	5'-GGAACAGTGAGGTGCCAGTAACG-3'
*PGC1α*	Mouse	5'-AAACTTGCTAGCGGTCCTCA-3'	5'-TGGCTGGTGCCAGTAAGAG-3'
*TFAM*	Mouse	5'-TTGGCTGGCTAAGCTCATCT-3'	5'-AAGGCTGAGAAGCGATAGCA-3'

### Mitochondrial Respiration Rate Analysis

A high-resolution respirometer (Oxygraphy2k, Oroboros Instruments GmbH) was equipped with a two-channel fluorescence optical module to simultaneously monitor oxygen concentration and H_2_O_2_ production as previously described (16, 17) with a data collection interval of 0.2 s. H_2_O_2_ release was measured from the fluorescence of resorufin formed from Amplex UltraRed reacting with H_2_O_2_ in the presence of horseradish peroxidase. Oxygen polarography was performed at 37°C in O2k-chambers. Oxygen concentration and oxygen flux per tissue mass were recorded real-time using DatLab software. Malate (0.5 mM) and glutamate (10 mM), ADP (2.5 mM), succinate (10 mM), retenone (10 μM) and antamycin (5 μM) were added into the chambers sequentially.

### Isolation of Cardiomyocytes and Mitochondrial Superoxide Measurements

At the end of protocol, mice were injected with 1,000 u/kg of heparin IV for anticoagulation before sacrifice (18). The heart was excised, cannulated and connected to a heart perfusion apparatus (Radnoti, CA) and perfusion was initiated in the Langendorff mode. The heart was perfused with a Ca^2+^- free based buffer (pH 7.2, 37^o^C) containing: 135 mM NaCl, 4 mM KCl, 1 mM MgCl_2_, 10 mM HEPES, 0.33 mM NaH_2_PO_4_, 10 mM glucose, 10 mM 2,3-butanedione monoxime (Sigma; B0753), and 5 mM taurine (Sigma; T0625) and bubbled with 95% O_2_/5% CO_2_. After 3–5 min of perfusion, the buffer was replaced with similar buffer containing 0.3 mg/g body weight collagenase D (Roche; 11088858001), 0.4 mg/g body weight collagenase B (Roche; 11088807001), and 0.05 mg/g body weight protease type XIV (Sigma; P5147) dissolved in 25 ml perfusion buffer (19). After complete digestion of the heart (6–8 min), the heart was removed and minced. The dispersed myocytes were then filtered through a 250 μm mesh cell collector. Cells were allowed to sediment by gravity.

MitoSOX Red (Invitrogen,M36008) was used to measure the production of mitochondrial superoxide, one of the reactive oxygen species (ROS). Isolated cardiomyocytes were loaded with MitoSOX Red (5 μM) in modified K-H buffer as described above for 10 min at 37°C protected from light, followed by wash out. Images were obtained of cardiomyocytes by use of fluorescence microscopes (excitation at 514 nm and measuring the emitted light at 585 nm).

### Statistical Analysis

Data were expressed as mean ± standard error of the means (SEM). Two-tailed Student's *t* test was used for two-group comparisons, while one-way or two-way ANOVA with Tukey's *post hoc* tests were used for multiple group comparisons (Prism 7.0, GraphPad Software, La Jolla, CA). A *p*-value of < 0.05 was considered statistically significant.

## Results

### EMPA Attenuated Cardiac Remodeling After TAC Without Changing Fasting Blood Glucose

**Figure 1:**
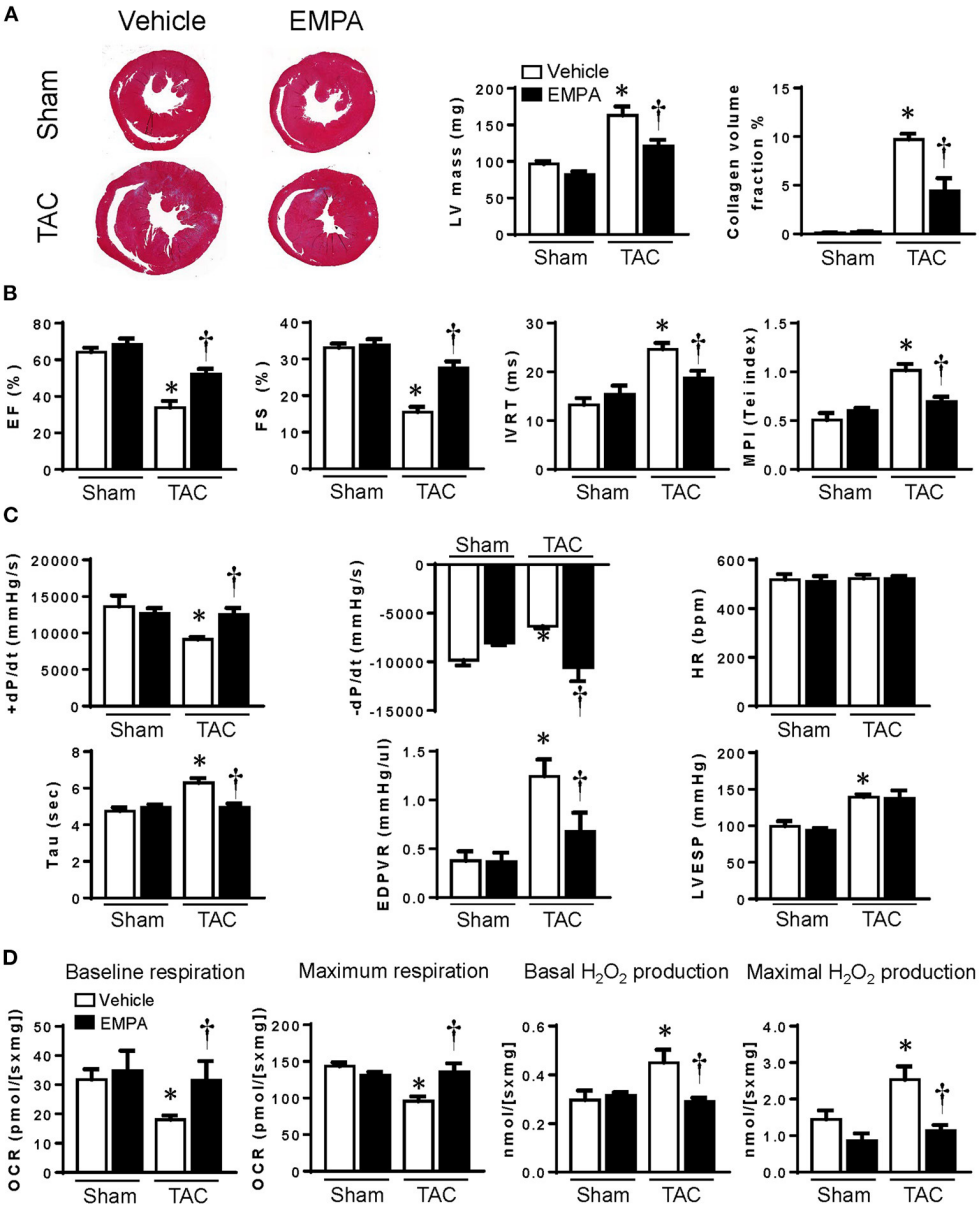
EMPA treatment attenuated remodeling of the LV, improved cardiac function and increased mitochondrial respiration. **(A)** Left, Masson staining of cross sections of hearts and Right (the collagen fibers were stained blue), quantitative analysis of left ventricle (LV) mass and heart collagen volume. **(B)** Transthoracic echocardiography showed the ejection fraction (EF), fraction shortening (FS), isovolumetric relaxation time (IVRT) and myocardial performance index (MPI) of hearts from different groups. **(C)** Cardiac function assessed by direct intraventricular pressure measurements. **(D)** Mitochondrial respiration and peroxide production in hearts from different groups. Original magnification × 100, Results are expressed as mean ± SEM, *n* = 5–7, *p < 0.05 vs. corresponding sham group, ^†^*p* < 0.05 vs. corresponding TAC vehicle group. One-way ANOVA and Tukey *post hoc* test. EMPA, empagliflozin; SEM, standard error of the mean; TAC, transverse aortic constriction; LV, left ventricle; HR, heart rate; EDPVR, end diastolic pressure volume relationship; LVESP, left ventricular end-systolic pressure; OCR, oxygen consumption rate.

Consistent with our previous findings, EMPA did not alter fasting blood glucose (FBG) or body weight in any of the different groups of mice (Table 2) (7). However, EMPA significantly attenuated TAC-induced cardiac hypertrophy (Figure 1A, left panel). Left ventricular (LV) mass increased by 68.9% after TAC and this increase was substantially attenuated by EMPA (Figure 1A, middle panel). TAC-induced increases in collagen deposition, indicated by Masson staining, were reduced by 54.2% with EMPA treatment (Figure 1A, right panel).

**Table 2:** Body weight and fasting blood glucose in different groups.

	**Sham**	**Sham + EMPA**	**TAC**	**TAC + EMPA**
**Body weight (g)**				
Baseline	27.7 ± 0.5	27.1 ± 0.4	29.6 ± 1.2	28.1 ± 0.8
6 weeks	29.4 ± 0.4	28.2 ± 0.5	30.1 ± 0.8	28.0 ± 1.0
**FBG (mg/dL)**				
Baseline	91.7 ± 4.2	106.2 ± 8.2	94.3 ± 6.2	89.9 ± 3.4
6 weeks	98.8 ± 8.6	101.7 ± 6.0	115.5 ± 5.1	107.7 ± 9.7

*Results are expressed as mean ± SEM, n = 5–7. One-way ANOVA and Tukey post hoc test. FBG, fasting blood glucose; EMPA, empagliflozin; TAC, transverse aortic constriction; SEM, standard error of the mean*.

### EMPA Attenuated Cardiac Systolic and Diastolic Dysfunction After TAC

Transthoracic echocardiography measurements of EF and FS were reduced 55.3% and 60.1%, respectively, after TAC and these changes were markedly attenuated by EMPA treatment, suggesting improvements of cardiac systolic function. TAC caused 85.9% and 100.6% elevations in IVRT and MPI, respectively, compared to the sham group and these changes were also largely reversed in mice treated with EMPA (Figure 1B).

In order to directly measure ventricular pressure and cardiac function *in vivo*, we inserted a Millar catheter into the LV though the apex. We assessed cardiac systolic function in these mice by LV pressure at end systole (LVESP) and +dP/dt_max_. Diastolic function was accessed by –dP/dt_max_ and Tau and ventricular stiffness by end diastolic pressure volume relationship (EDPVR). Although EMPA did not reduce the elevation in LVESP after TAC, it markedly reduced Tau and EDPVR, suggesting improvement of diastolic function and increased compliance of the LV (Figure 1C).

### EMPA Reduced Intracellular ROS and Increased Endogenous Antioxidant After TAC

We measured oxygen consumption and H_2_O_2_ production in LV muscle fibers from different groups (Figure 1D). The results showed that both basal and maximum oxygen consumption rates (OCR) were reduced after TAC, while basal and maximum H_2_O_2_ production were increased. However, EMPA treatment increased OCR and attenuated H_2_O_2_ production at basal and maximal conditions after TAC.

**Figure 2:**
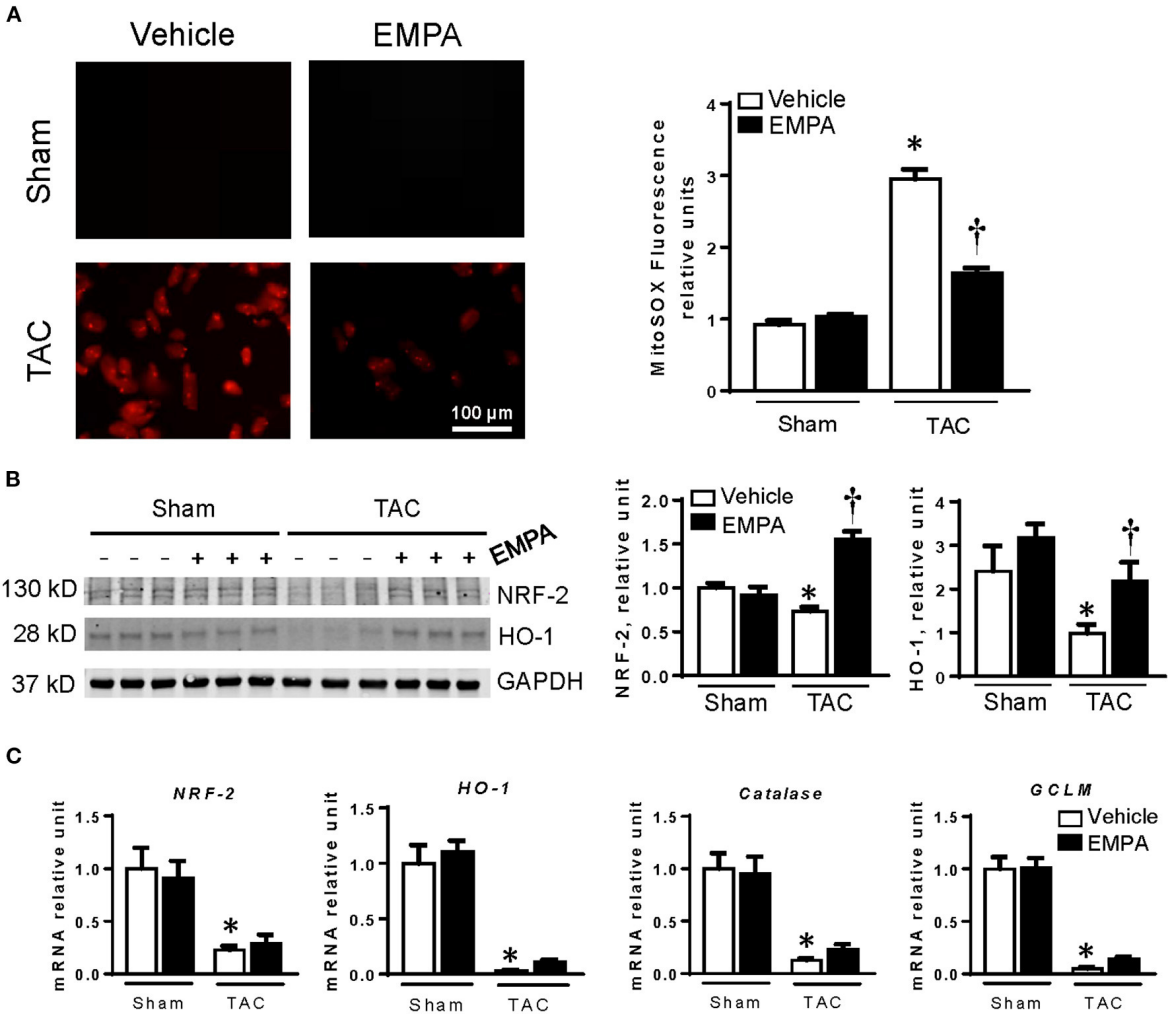
EMPA treatment attenuated cardiomyocyte overproduction of reactive oxygen species (ROS) induced by TAC. **(A)** Left, Mitosox staining of cardiomyocytes in sham, sham + EMPA, TAC and TAC + EMPA groups and Right, quantitative results. **(B)** Representative blots of NRF-2 and HO-1, and quantitative results. **(C)** The relative mRNA levels of genes related to endogenous antioxidants. Results are expressed as mean ± SEM, *n* = 5–7, *p < 0.05 vs. corresponding sham group, ^†^*p* < 0.05 vs. corresponding TAC vehicle group. One-way ANOVA and Tukey *post hoc* test. EMPA, empagliflozin; SEM, standard error of the mean; TAC, transverse aortic constriction; NRF-2, the nuclear factor erythroid 2–related factor 2; HO-1, heme oxygenase-1; GCLM, glutamate-cysteine ligase modifier subunit; GAPDH, glyceraldehyde 3-phosphate dehydrogenase.

To measure production of superoxide, isolated cardiomyocytes were stained with Mitosox red. After 6 weeks of TAC, cardiomyocyte production of superoxide was significantly increased compared to sham group, however, EMPA treatment reduced superoxide in these cells by 44.4% (Figure 2A).

Cardiac expression of endogenous antioxidant-related genes was significantly reduced after TAC (Figures 2B,C). Protein and mRNA levels of NRF-2 and HO-1 were decreased after TAC. Also, the important antioxidant catalase and glutamate-cysteine ligase modifier subunit (GCLM) were downregulated in hearts of the TAC group. EMPA treatment restored cardiac expression of HO-1 and NRF-2 proteins after TAC. However, the mRNA levels of NRF-2, HO-1, catalase and GCLM were not changed after EMPA treatment.

### EMPA Enhanced Mitochondrial Biogenesis After TAC

**Figure 3:**
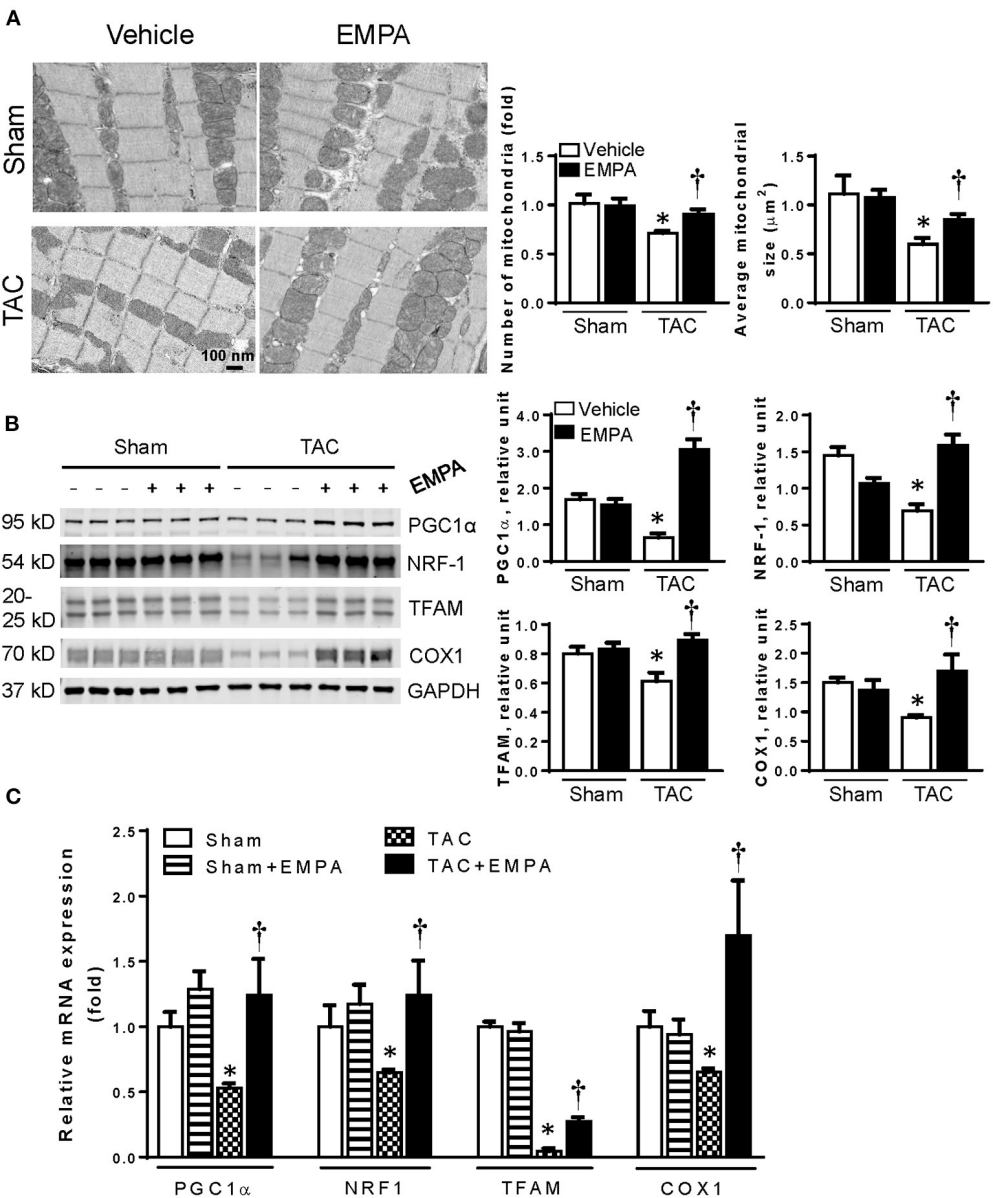
EMPA treatment increased mitochondrial biogenesis. **(A)** Left, Transmission electron microscopy showing the morphology of mitochondria in sham, sham + EMPA, TAC and TAC + EMPA groups and Right, quantitative analysis of mitochondrial size and counts. Results are expressed as mean ± SEM, *n* = 3–5, *p < 0.05 vs. corresponding sham, ^†^*p* < 0.05 vs. corresponding TAC. **(B)** Left, Representative blots of mitochondrial biogenesis-related proteins and Right, quantitative results. **(C)** Relative mRNA levels of PGC1α, NRF1, TFAM and COX1. Results are expressed as mean ± SEM, *n* = 5–7, *p < 0.05 vs. corresponding sham group, ^†^*p* < 0.05 vs. corresponding TAC vehicle group. One-way ANOVA and Tukey *post hoc* test. EMPA, empagliflozin; SEM, standard error of the mean; TAC, transverse aortic constriction; PGC1-α, peroxisome proliferator-activated receptor gamma coactivator 1-alpha; NRF-1, nuclear respiratory factor 1; TFAM, mitochondrial transcription factor A; COX1, cyclooxygenase1; GAPDH, glyceraldehyde 3-phosphate dehydrogenase.

TEM clearly showed that mitochondrial number in LV cardiomyocytes was significantly reduced after TAC compared to the sham group (Figure 3A). Moreover, the mean mitochondrial area was also significantly smaller than in the sham group. EMPA treatment in TAC mice increased cardiomyocyte mitochondrial number and mean area (Figure 3A).

To determine how EMPA affected mitochondrial biogenesis in hearts after TAC, the expressions of PGC1α, NRF-1 and TFAM, which are involved in mitochondrial biogenesis, were investigated by immunoblotting and RT-qPCR (Figures 3B,C). The results showed that EMPA treatment increased expression of PGC1α, NRF-1 and TFAM after TAC. Furthermore, EMPA increased expression of COX1, also suggesting that EMPA increased mitochondrial count in TAC mice.

### EMPA Reduced Apoptosis and Cyt C Release From Mitochondria to Cytosol After TAC

**Figure 4:**
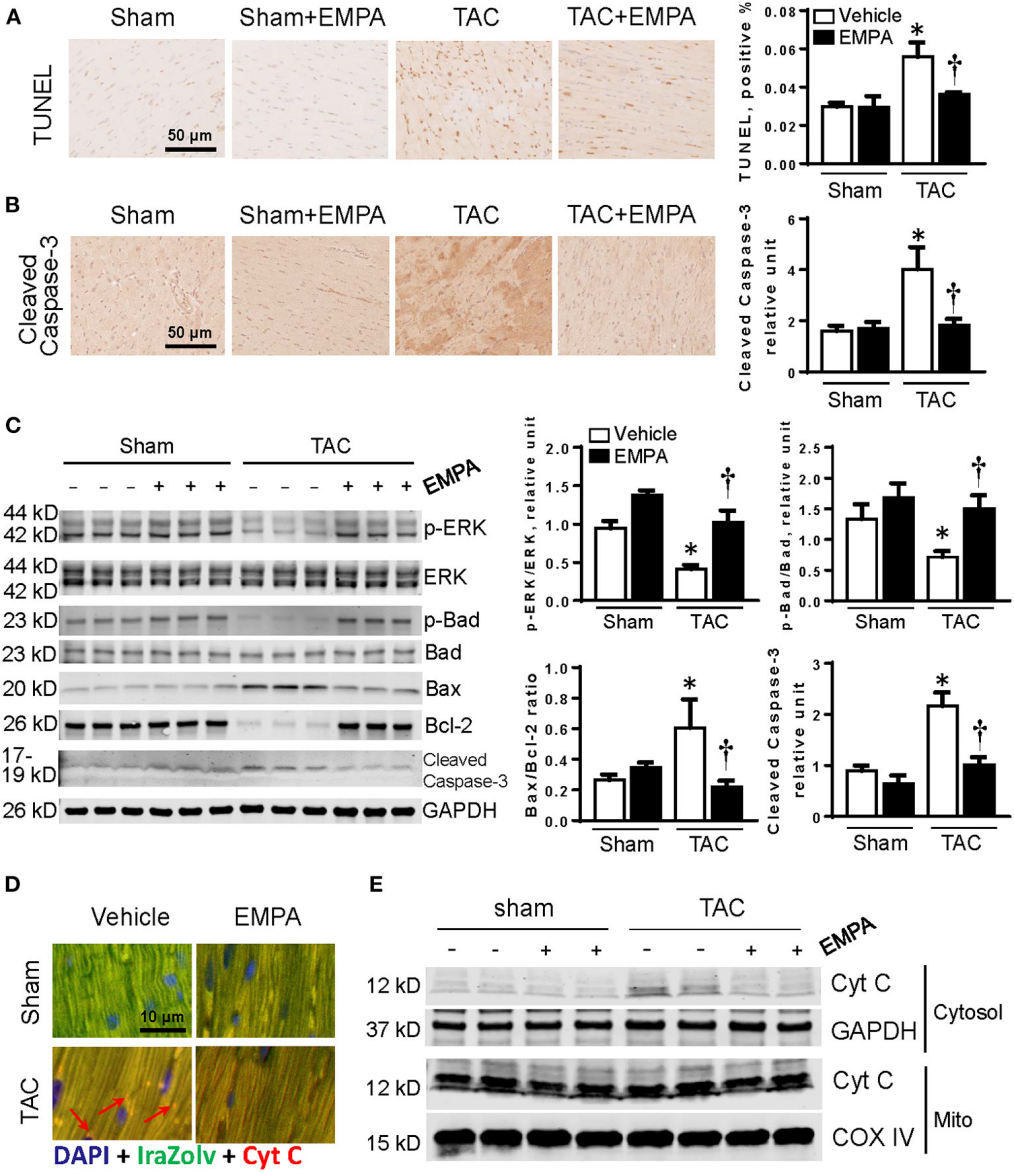
EMPA treatment attenuated apoptosis in TAC-induced HF. **(A)** TUNEL staining of heart sections in sham, sham + EMPA, TAC and TAC + EMPA groups. **(B)** Immunohistochemistry results of cleaved caspase-3 in different groups. **(C)** Left, representative blots of proteins related to cellular survival and apoptotic pathways and Right, quantitative results. **(D,E)** Increased Cyt C released from mitochondria into cytoplasm after TAC is attenuated by EMPA treatment. Results are expressed as mean ± SEM, *n* = 5–7, *p < 0.05 vs. corresponding sham group, ^†^*p* < 0.05 vs. corresponding TAC vehicle group. One-way ANOVA and Tukey *post hoc* test. EMPA, empagliflozin; SEM, standard error of the mean; TAC, transverse aortic constriction; ERK, extracellular signal-regulated kinase; Cyt C, cytochrome C; COX IV, cytochrome c oxidase, complex IV in the mitochondrial respiratory chain; DAPI, 4',6-diamidino-2-phenylindole; GAPDH, glyceraldehyde 3-phosphate dehydrogenase.

The number of TUNEL positive cells as well as expression of cleaved caspase-3 in the LV were significantly increased after TAC indicating increased apoptosis. However, EMPA treatment attenuated the percentage of TUNEL positive cells and expression of cleaved caspase-3 in LVs of TAC mice (Figures 4A,B). To investigate how EMPA regulates cardiac apoptosis, the ERK-Bad pathway was assessed by immunoblotting. As shown in Figure 4C, TAC reduced phosphorylation of ERK and Bad, Bcl-2, but increased Bax and cleaved caspase-3. Conversely, EMPA restored the p-ERK/ERK and p-Bad/bad, reduced cleaved caspase-3 and Bax/Bcl-2 ratio.

Due to mitochondrial dysfunction, mitochondrial outer membrane permeabilization may be changed (20). Thus we separated the mitochondria from cytosol, and found that Cyt C was released from cardiac mitochondria into the cytosol in the TAC group. However, EMPA reduced the release of Cyt C from mitochondria after TAC (Figures 4D,E).

### EMPA Improved Autophagy in Hearts After TAC

**Figure 5:**
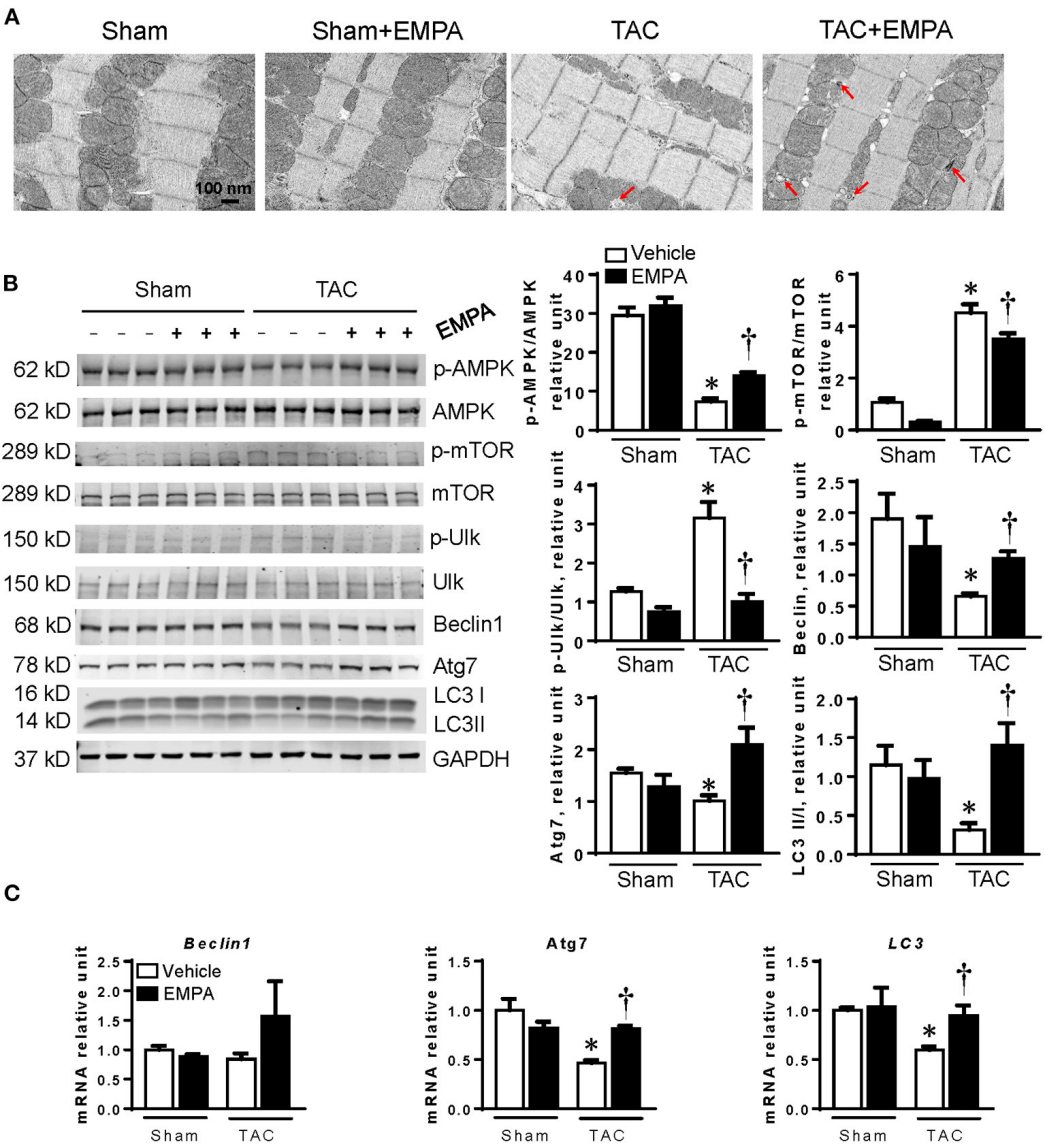
EMPA treatment enhanced autophagy in failing hearts. **(A)** Transmission electron microscopy (TEM) showed autophagosomes in the hearts. Red arrows point to autophagosomes. **(B)** EMPA activated autophagy pathway by inhibiting mTOR pathway. **(C)** RT-PCR results showed that the mRNA expression of Beclin1, Atg7 and LC3 were increased by EMPA treatment after TAC. Results are expressed as mean ± SEM, *n* = 5–7, *p < 0.05 vs. corresponding sham group, ^†^*p* < 0.05 vs. corresponding TAC vehicle group. One-way ANOVA and Tukey *post hoc* test. EMPA, empagliflozin; SEM, standard error of the mean; TAC, transverse aortic constriction; AMPK, AMP-activated protein kinase; mTOR, mammalian target of rapamycin; Ulk, Unc-51 like autophagy activating kinase; Atg7, autophagy related 7; LC3, light chain 3; GAPDH, glyceraldehyde 3-phosphate dehydrogenase.

TEM images showed that the number of autophagosomes in the TAC + EMPA group was larger than in other groups (Figure 5A). Additionally, the autophagy-related signal pathway was investigated by immunoblotting. The results showed that phosphorylation of AMPK, mTOR and Ulk was reduced after TAC, but restored by EMPA treatment. Also, the Beclin1, Atg7 and ratio of LC3 II/I were decreased after TAC. However, EMPA treatment increased cardiac expression of Beclin1 and Atg7, and increased the LC3 II/I ratio. Furthermore, RT-qPCR results showed that the mRNA levels of Atg7 and LC3 were reduced after TAC but increased by EMPA treatment. However, the mRNA level of Beclin1 was not different among these groups (Figures 5B,C).

### Acute EMPA Treatment Increased Cardiac Mitochondrial Respiration and Reduced Hydrogen Peroxide Production in Isolated Cardiac Muscle Fibers From Failing Hearts

**Figure 6:**
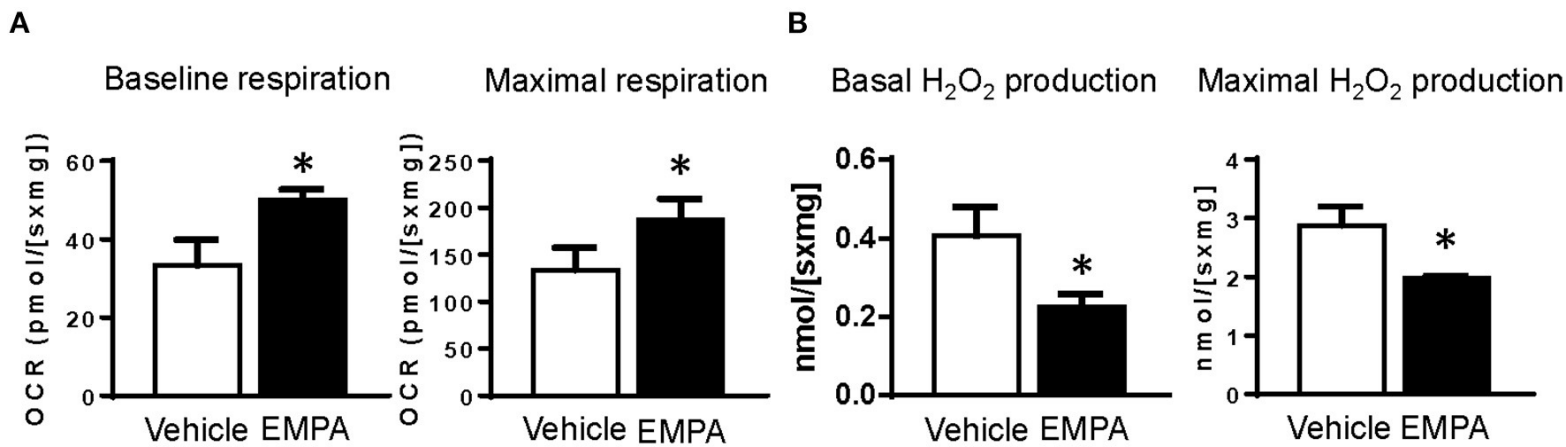
Acute treatment of hearts from TAC groups with EMPA improved mitochondrial function. **(A)** Baseline and maximal oxygen consumption rate in heart tissues treated with vehicle or EMPA. **(B)** Baseline and maximal peroxide production in heart tissues treated with vehicle or EMPA. Results are expressed as mean ± SEM, *n* = 7, *p < 0.05 vs. vehicle group. Student *t* test. EMPA, empagliflozin; SEM, standard error of the mean; TAC, transverse aortic constriction; OCR, oxygen consumption rate.

To investigate whether EMPA has direct effects on the heart, we divided the muscle fibers of the LV from the TAC group into two parts: tissues treated with EMPA (1.0 mM) or vehicle for 1 h before placing them into the Oxygraphy2K chambers with EMPA (1.0 mM) or vehicle. The results showed that acute EMPA treatment, similar to the effects of chronic treatment, significantly increased baseline and maximal OCR, and reduced basal and maximal peroxide production compared to vehicle groups (Figures 6A,B).

## Discussion

The beneficial effects of SGLT2 inhibitors in treating HF have been documented in several clinical trials (2, 21). However, the mechanisms by which SGLT2 inhibitors protect the heart in HF are still controversial. Although our previous study demonstrated that EMPA, a highly selective SGLT2 inhibitor, may have direct effects in the heart to improve glucose and fatty acid oxidation and increase ATP production in HF (7), the mechanisms involved remain unclear. To further investigate how EMPA regulates cardiac metabolism, we focused on the mitochondria since they are the “powerhouses” of the cells and HF is associated with impaired mitochondrial function. Our results indicate that EMPA can enhance mitochondrial function by upregulating mitochondrial biogenesis, enhance OXPHOS, reduce ROS production, attenuate apoptosis, and increase autophagy to improve overall cardiac function in pressure overload-induced HF.

### EMPA Treatment Attenuated LV Hypertrophy and Fibrosis After TAC

Cardiac hypertrophy and fibrosis are independent risk factors for development of HF (22). Our results, in line with others, showed that SGLT2 inhibitors can induce LV mass regression in HF (23, 24). Although Byrne et al. reported that EMPA prevents worsening of cardiac function induced by TAC (8), they did not find obvious improvement of cardiomyocyte hypertrophy after EMPA treatment. One possible mechanism that has been proposed for the anti-hypertrophic effect of SGLT2 inhibitors is the blood pressure-lowering effect (25). However, this effect is mild. We did not observe obvious reductions in intraventricular or systemic pressure in TAC + EMPA groups compared to the TAC group. It is known that mTORC1 plays an important role in protein synthesis, cellular proliferation and metabolism (26, 27), and prolonged and excessive activation of mTORC1 can cause cardiac hypertrophy (28). EMPA may inhibit mTORC1's activity through AMPK (the mechanism by which EMPA activates AMPK will be discussed later), preventing the formation of the complex (29, 30). In addition, EMPA can upregulate expression of Sestrin2, a stress-induced protein, which can directly inhibit activation of mTORC1. Thus, EMPA may attenuate cardiac hypertrophy by inhibiting mTORC1 to decrease protein synthesis in hearts.

EMPA also reduced cardiac fibrosis in TAC-induced HF. Other SGLT2 inhibitors, e.g. dapagliflozin and canagliflozin, can also reduce tissue fibrosis (31). Since SGLT2 is mainly expressed in kidneys, but not in cardiomyocytes or cardiac fibroblasts (32), SGLT2 inhibitors may reduce myocardial fibrosis by pleiotropic effects. However, there is evidence for anti-fibrotic effects of SGLT2 inhibitors in diabetic rats (33). Oxidative stress/excessive ROS can induce profibrotic factors such as transforming growth factor β (TGF-β) and increase proliferation of myofibroblasts (34). Thus, SGLT2 inhibitors may attenuate cardiac fibrosis by reducing ROS production and restoration of redox homeostasis in failing hearts (34).

### EMPA Treatment Improved Mitochondrial Biogenesis and Reduced ROS Generation in TAC-Induced HF

An important finding of the present study is that EMPA treatment for four weeks significantly increased mitochondrial numbers and reduced cellular ROS in hearts exposed to chronic TAC. One potential mechanism of improved mitochondrial biogenesis is activation of the PGC1α/NRF-1 pathway by EMPA. PGC1α is a transcriptional coactivator that is considered to be a critical inducer of mitochondrial biogenesis in cells (35, 36). SGLT2 inhibitors treatment may induce a fasting-like paradigm, leading to the activation of PGC1α pathway (37). Our results are consistent with this hypothesis since EMPA treatment substantially increased cardiac expression of PGC1α and NRF-1 in TAC-induced HF.

The anti-oxidation effects of EMPA are impressive. Both basal and maximum H_2_O_2_ production were significantly reduced by chronic EMPA treatment in failing hearts. Additionally, EMPA treatment decreased superoxide in cardiomyocytes from TAC groups.

To investigate how EMPA regulates endogenous antioxidants, the NRF-2/HO-1 pathway was evaluated. NRF-2 is a transcription factor responsible for maintenance of cellular redox homeostasis and phase II detoxification responses in mammals (38), and NRF-2/antioxidant response element (ARE) signaling is an important antioxidant pathway involved in the defense against oxidative stress (35, 36). HO-1 is one of the ARE-regulated phase II detoxifying enzymes regulated by NRF-2, which provides robust protection against oxidative stresses (37).

Our results show that EMPA increased the protein levels of NRF-2 and HO-1 after TAC. However, EMPA did not change mRNA levels of NRF-2, HO-1, catalase and GCLM. These results suggest that EMPA may reduce degradation of these proteins rather than increase their translation, although the detailed mechanisms responsible for increased levels of NRF-2 and HO-1 are uncertain and require further investigation.

### EMPA Enhances Cardiac Autophagy and Reduces Apoptosis of Cardiomyocytes

Autophagy is an adaptive response of cells to stress. It can recycle damaged mitochondria and other organelles for the resynthesis of new organelles and ATP (39). Impaired autophagy increases susceptibility of the heart to stresses and promotes HF (40, 41). EMPA partially restored activity of AMPK and inhibited mTOR, which could then enhance autophagy in LV cardiomyocytes. Furthermore, autophagy also plays an important role in maintaining cellular ROS homeostasis by eliminating oxidized peroxisomes (42), which provides a clue to the mechanism by which EMPA attenuates ROS production in failing hearts.

Mitochondrial dysfunction and overproduced ROS can cause cellular apoptosis by increasing permeabilization of mitochondrial outer membranes and releasing Cyt C from mitochondria into the cytosol, thus activating caspase and apoptosis (43). However, we found that EMPA increased phosphorylation of ERK and Bad, upregulated Bcl-2, and reduced Bax expression in hearts exposed to TAC. It is known that activated ERK can phosphorylate Bad at Ser112 through p90 ribosomal S6 kinase (RSK) (44), and then convert Bcl-2 into an activated form, preventing pore formation in the mitochondrial outer membrane and Cyt C release, and as a result reducing cellular apoptosis (45). Thus, EMPA treatment may reduce Cyt C release and attenuate myocardial apoptosis in TAC-induced HF via the ERK/Bad pathway.

### EMPA Acts Directly on the Heart to Improve Mitochondrial Respiration

Although we demonstrated that EMPA can improve mitochondrial respiration in HF, there is still a question of whether the improvements of mitochondrial function by EMPA result from direct effects on the heart. As shown in Figure 5, incubating LV muscle fibers from TAC hearts with EMPA increased the OCR and reduced H_2_O_2_ production, indicating that EMPA has direct cardiac effects. Our previous study suggested that EMPA may directly bind glucose transporters, inhibit excessive glycolysis and improve glucose oxidation and fatty acid oxidation (7). In addition, we also demonstrated that EMPA can directly activate the AMPK pathway in the heart (6), and activated AMPK is known to enhance autophagy, reduce apoptosis, decrease ROS production and increase mitochondrial biogenesis (18, 46, 47). EMPA may activate AMPK through liver kinase B1 (LKB1) and/or by increasing Sestrin2 (6, 48). Furthermore, EMPA may suppress Drp1 phosphorylation at Ser616, and increase Drp1 phosphorylation at Ser637, leading to inhibition of mitochondrial fission by triggering the AMPK pathway, ultimately improving cardiac mitochondrial function (49).

### Limitations

Although the present study demonstrates that EMPA has multiple direct effects on cardiomyocytes that improve mitochondrial respiration and energy metabolism, our findings do not rule out the possibility that systemic effects of EMPA can also contribute to overall improvement of cardiac function in pressure overload-induced HF. Previous studies have provided evidence that multiple systemic effects of SGLT2 inhibition, including decreased blood pressure, natriuresis, glycosuria, decreased extracellular fluid volume, and improved kidney function, may provide beneficial cardiovascular effects in patients with HF, with or without diabetes (5, 50). However, observations from other studies (5, 50), coupled with our findings of improved cardiac mitochondrial function and energy metabolism in *ex vivo* cardiomyocytes, make it unlikely that all of EMPA's beneficial effects in the TAC-induced model of HF can be explained by reductions in blood pressure and other systemic actions.

Although all of the mice used in this study were male, our preliminary data showed that female mice exhibit similar results. Potential sex differences during EMPA treatment in HF should be further investigated in future studies.

## Conclusions and Perspectives

**Figure 7:**
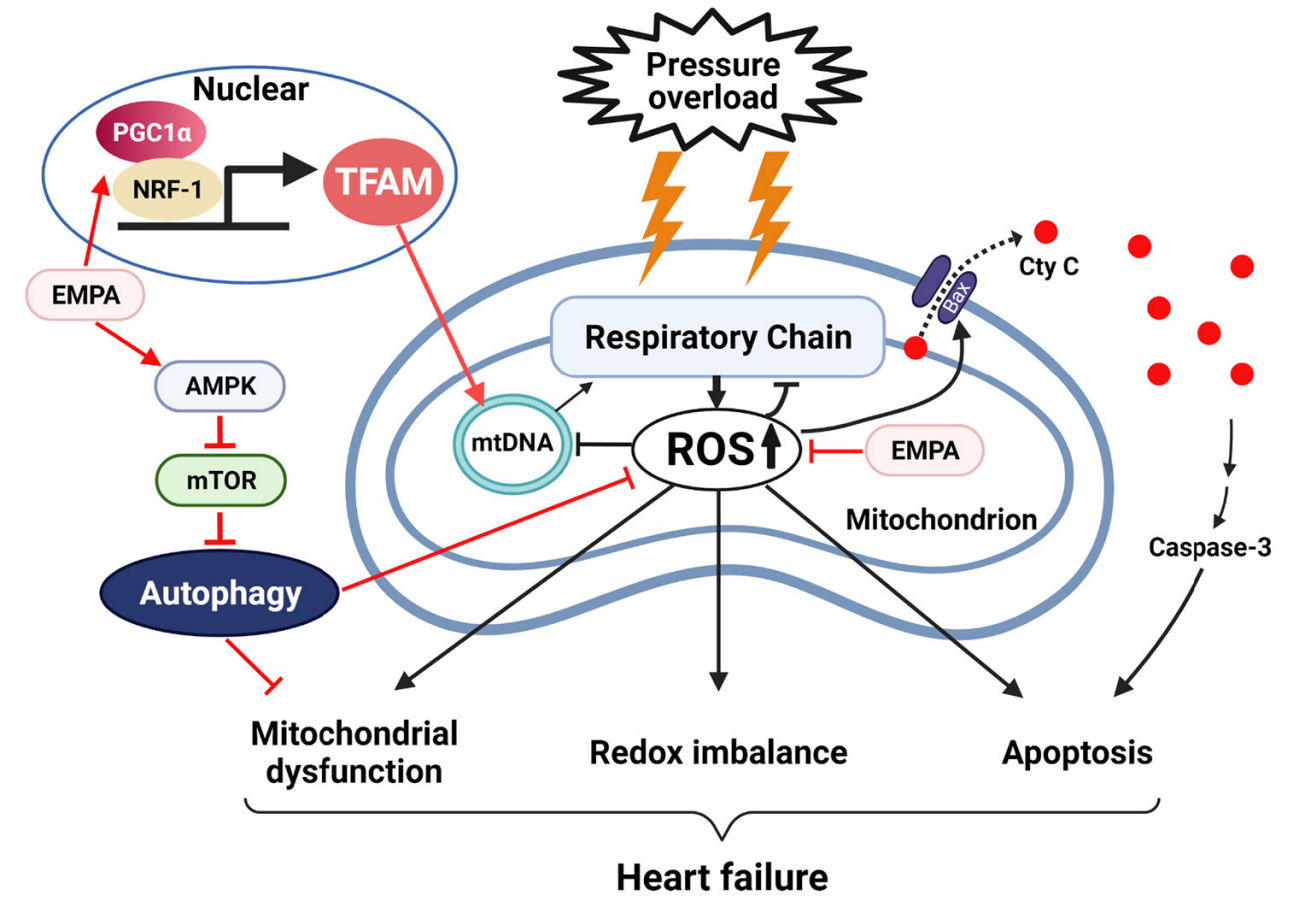
Empagliflozin improves mitochondrial function, restores redox homeostasis and reduces apoptosis in heart failure. EMPA, empagilflozin; NRF-1, nuclear respiratory factor 1; TFAM, mitochondrial transcription factor A; mtDNA, mitochondrial DNA; ROS, reactive oxygen species; AMPK, adenosine monophosphate-activated protein kinase; mTOR, mammalian target of rapamycin.

Our results indicate that SGLT2 inhibition with EMPA increases cardiac mitochondrial biogenesis, reduces ROS production, decreases myocardial apoptosis, enhances autophagy, and activates the AMPK-mTOR pathway, thus improving cardiomyocyte mitochondrial function after TAC-induced HF (Figure 7). These protective functions of EMPA occur by direct cardiac effects and contribute to improved systolic and diastolic function in a non-diabetic model of HF induced by pressure overload.

The mechanisms by which EMPA binds to cardiac cells and exerts its protective effects have not been fully elucidated. However, we recently provided evidence from molecular docking analysis and isolated perfused heart studies suggesting that EMPA can bind cardiac glucose transporters to reduce cardiac glycolysis and rebalance coupling between glycolysis and oxidative phosphorylation. These direct cardioprotective effects of EMPA, which improve mitochondrial function and occur independently of reductions in blood glucose or diuresis, highlight the potential beneficial effects SGLT2 inhibitors in other conditions associated with mitochondrial dysfunction, such in myocardial toxicity associated with chemotherapeutics.

## Data Availability Statement

The raw data supporting the conclusions of this article will be made available by the authors, without undue reservation.

## Ethics Statement

The animal study was reviewed and approved by University of Mississippi Medical Center.

## Author Contributions

XL and JH designed the study, conducted the research, and wrote the article. EF, JC, ZW, AS, AM, AO, and MH contributed to the revision of the article for important content, prepared the figures, and helped to analyze and interpret the data. All authors have read and approved the final version of the article.

## Funding

This work was supported by the National Heart, Lung, and Blood Institute of the National Institutes of Health (NIH) [Grant Number P01HL051971], the National Institute of General Medical Sciences [Grant Numbers P20GM104357 and U54GM115428], the National Institute of Diabetes and Digestive and Kidney Diseases [Grant Numbers R01 DK121411, R01 DK121748, and R00 DK113280], and American Heart Association [Grant Number 908950].

## Conflict of Interest

The authors declare that the research was conducted in the absence of any commercial or financial relationships that could be construed as a potential conflict of interest.

## Publisher's Note

All claims expressed in this article are solely those of the authors and do not necessarily represent those of their affiliated organizations, or those of the publisher, the editors and the reviewers. Any product that may be evaluated in this article, or claim that may be made by its manufacturer, is not guaranteed or endorsed by the publisher.
